# EgoSurgery‐HTS: A Dataset for Egocentric Hand–Tool Segmentation in Open Surgery Videos

**DOI:** 10.1049/htl2.70049

**Published:** 2025-12-09

**Authors:** Nathan Darjana, Ryo Fujii, Hideo Saito, Hiroki Kajita

**Affiliations:** ^1^ Faculty of Science and Technology Keio University Yokohama Japan; ^2^ Department of Plastic and Reconstructive Surgery Keio University School of Medicine Tokyo Japan

**Keywords:** computer vision, medical image processing, object detection

## Abstract

Egocentric open‐surgery videos capture rich, fine‐grained details essential for accurately modelling surgical procedures and human behaviour in the operating room. A detailed, pixel‐level understanding of hands and surgical tools is crucial for interpreting a surgeon action and intention. We introduce EgoSurgery‐HTS, a new dataset with pixel‐wise annotations and a benchmark suite for segmenting surgical tools, hands, and interacting tools in egocentric open‐surgery videos. Specifically, we provide a labelled dataset for (1) tool instance segmentation of 14 distinct surgical tools, (2) hand instance segmentation, and (3) hand–tool segmentation to label main operating hands and the tools they manipulate. Using EgoSurgery‐HTS, we conduct extensive evaluations of state‐of‐the‐art segmentation methods and demonstrate significant improvements in the accuracy of hand and hand–tool segmentation in egocentric open‐surgery videos compared to existing datasets. The dataset will be released upon acceptance.

## Graphical abstract

We present EgoSurgery‐HTS, a new egocentric open‐surgery video dataset with pixel‐level annotations for surgical tool, hand, and hand–tool interaction segmentation. It includes instance‐level labels for 14 surgical tools and operating hands, enabling fine‐grained modelling of surgical actions and intentions. Benchmarking with state‐of‐the‐art methods shows significant improvements in segmentation accuracy over existing datasets; the dataset will be released upon acceptance.

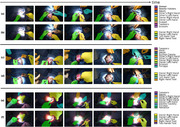

## 1 Introduction

The automated analysis of egocentric open‐surgery videos plays an important role in applications such as real‐time surgical assistance, skill assessment [1], and medical procedure evaluation.

By providing fine‐grained procedural insights, automated analysis has the potential to enhance surgical precision, reduce operative duration, and improve patient outcomes. A key aspect of this analysis is surgical scene segmentation, which enables per‐pixel understanding of the operative field. In particular, the segmentation of hands and surgical tools is essential for interpreting a surgeon's actions and intent, facilitating workflow optimization and AI‐assisted surgery.

Surgical tool segmentation, which involves accurately identifying and delineating instruments during procedures, has been extensively studied in the context of minimally invasive surgery (MIS). This progress has been fueled by the availability of large, well‐annotated datasets [6, 7, 9], enabling the development of advanced segmentation methods [10, 11, 12, 13, 14, 15, 16, 17]. However, these datasets and methods are tailored to MIS, where instruments are manipulated within narrow visual fields, under controlled lighting, and from fixed viewpoints. In contrast, tool segmentation in open surgery remains largely underexplored. Open procedures present unique challenges, including the simultaneous manipulation of multiple instruments by several individuals, greater variability in camera viewpoints, and frequent changes in lighting conditions. The lack of large‐scale, domain‐specific datasets for open surgery significantly limits progress in this direction.

Hand segmentation is equally important for open surgery video understanding, as surgeons' hands are central to almost every frame, guiding instrument manipulation and reflecting surgical workflow. While hand segmentation has been studied in non‐surgical contexts [18, 19], these datasets do not generalize to surgery due to substantial differences in appearance and motion [20]. Moreover, the segmentation of main hands, in first‐person camera view, together with interacting objects, which are commonly studied in egocentric daily activity datasets [21, 22, 23, 24, 25], has received little attention in open surgical settings, despite being critical for capturing the primary surgical actions performed by the operating surgeon.

Building upon the EgoSurgery dataset [26, 27, 28] and leveraging its comprehensive annotation suite for open surgery video understanding, we introduce EgoSurgery‐HTS, a novel and detailed dataset designed to facilitate pixel‐level analysis of open surgery scenes. Firstly, EgoSurgery‐HTS provides tool instance segmentation annotations, offering fine‐grained segmentation masks across 14 distinct types of surgical tools. Secondly, it includes segmentation annotations for four types of hand instances. Finally, EgoSurgery‐HTS features main hands‐object segmentation annotations, providing fine‐grained per‐pixel labels for main hands and their interacting tools. Using the proposed EgoSurgery‐HTS dataset, we conduct a systematic study on mainstream segmentation baselines. Furthermore, with this new dataset, we significantly improve hand and hand‐object segmentation performance compared to previous datasets in the open surgery domain, demonstrating the value and impact of EgoSurgery‐HTS in advancing open surgery video analysis. Table 1 shows a comparison of EgoSurgery‐HTS with existing surgical segmentation datasets.

**TABLE 1:** Comparison of EgoSurgery‐HTS with existing surgical segmentation datasets.

Dataset	Surgery type	Frames	Tool types	Hand types	Hand‐tool onteraction
Endovis2015 [2]	MIS	10K	3	—	×
Endovis2017 [3]		2.4K	10	—	×
Endovis2018 [4]		2.4K	10	—	×
CholecSeg8k [5]		8K	12	—	×
AutoLaparo [6]		1.8K	7	—	×
ROBUSTMIS2019 [7]		10K	2	—	×
SAR‐RARP50 [8]		10K	10	—	×
Grey EgoSurgery‐HTS (ours)	Open surgery	18.4K	14	4	✓

Our main contributions are summarized as follows: (1) We introduce EgoSurgery‐HTS, a comprehensive dataset tailored for fine‐grained understanding of egocentric open surgery. It includes detailed annotations for tool instance segmentation, hand instance segmentation, and hand‐object segmentation, enabling advanced analysis of complex surgical scenes. (2) We conduct extensive evaluations of state‐of‐the‐art instance segmentation methods for each task on EgoSurgery‐HTS, and discuss their strengths and weaknesses. (3) Models trained on EgoSurgery‐HTS demonstrate superior performance in hand and hand‐object segmentation, significantly outperforming models trained on pre‐existing datasets.

## 2 Dataset

### 2.1 Dataset Source and Annotations

The EgoSurgery dataset [26, 27, 28] comprises 21 videos spanning 10 distinct surgical procedures, with a total duration of 15 h, performed by 8 surgeons. EgoSurgery provides over 27K frames with phase annotations and 19K6 frames with bounding box annotations for surgical tools and hands. The 20 EgoSurgery videos were originally recorded at 25 fps with a resolution of 1920 × 1080 pixels. To ensure anonymity, they are provided at a subsampled rate of 0.5 fps, with faces blurred. However, EgoSurgery lacks per‐pixel segmentation labels for surgical tools, hands, and their interactions. Therefore, we introduce EgoSurgery‐HTS, an extension of EgoSurgery that includes additional annotations for surgical tool segmentation, hand segmentation, and hand–tool segmentation on a subset of the existing dataset. These comprehensive annotations establish EgoSurgery as the only available dataset enabling multi‐task learning for phase recognition, surgical tool detection, hand detection, surgical tool segmentation, hand segmentation, and hand–tool segmentation.

#### 2.1.1 Annotation Process

**FIGURE 1:**
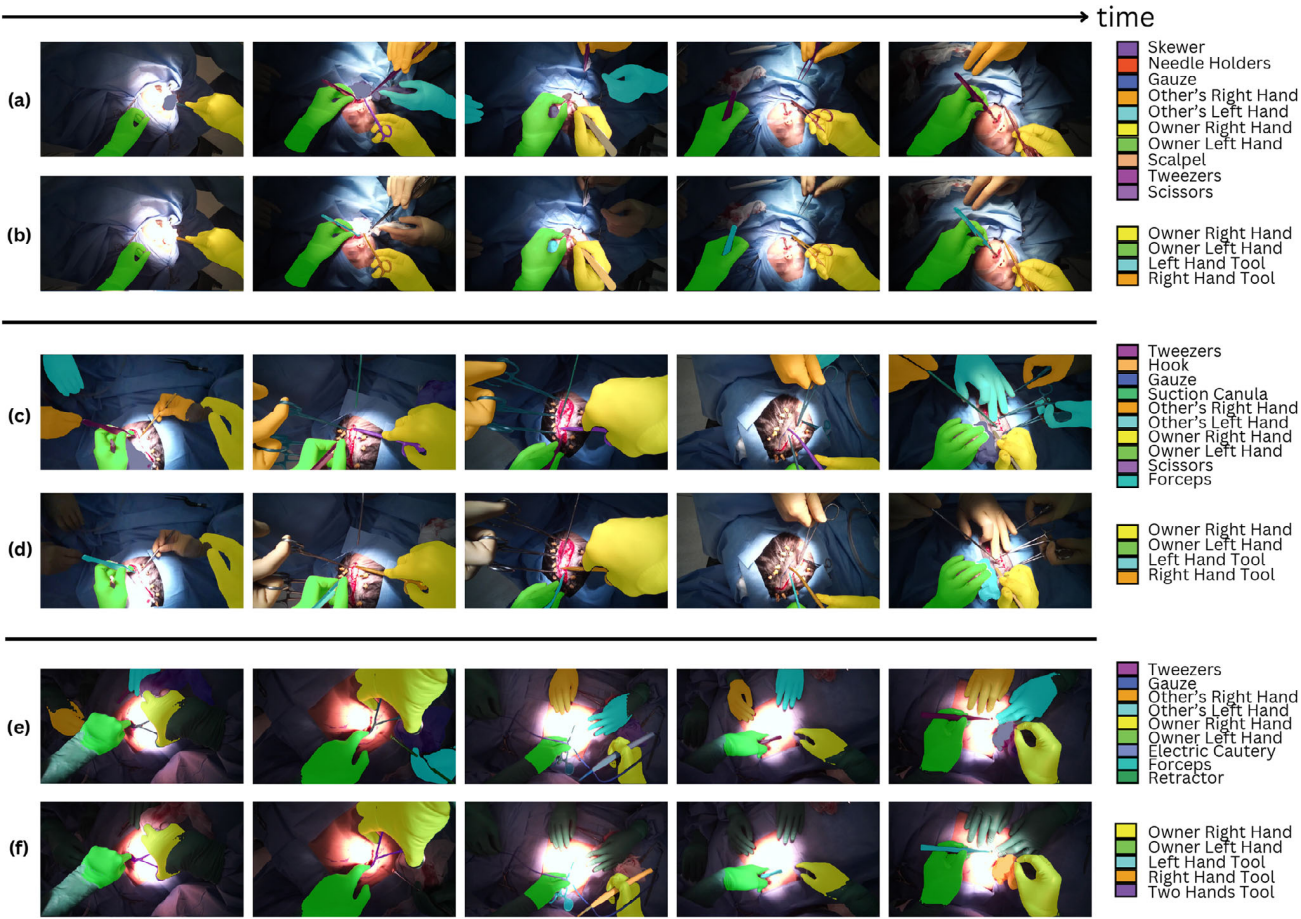
Overview of sparse segmentation from three different videos, showing all segmented tool and hand instances (top) and, separately, only the main hand–tool interactions (bottom). (a), (c), (e) Overview of every tool and hand instance segmentation task. (b), (d), (f) Overview of hand‐object segmentation task.

Inspired by SAMRS [29], which leverages SAM [30] and existing remote sensing object detection datasets to construct a large‐scale remote sensing segmentation dataset, we apply SAM to EgoSurgery, using tool and hand bounding box annotations to generate segmentation labels. For hand–tool segmentation annotations, interacting tool annotations are determined by selecting the tool with the highest IoU score relative to the hand segmentation. For each image in the dataset, we obtain segmentation masks for 14 types of surgical tools, along with per‐pixel hand mask annotations where applicable. These hand‐related masks include: (a) own left hand, (b) own right hand, (c) other left hand, and (d) other right hand. Additionally, we provide hand–tool segmentation masks, corresponding to the main operating hands and their associated tools, categorized as: (a) left‐hand, (b) right‐hand, (c) left‐hand object, (d) right‐hand object, and (e) two‐hand object. All generated annotations undergo manual review and correction to ensure accuracy. An overview of the generated dataset is visible in Figure 1. This figure highlights the distinction between segmenting all hand and tool instances in the images versus segmenting only the primary operating hand together with its associated tools.

**FIGURE 2:**
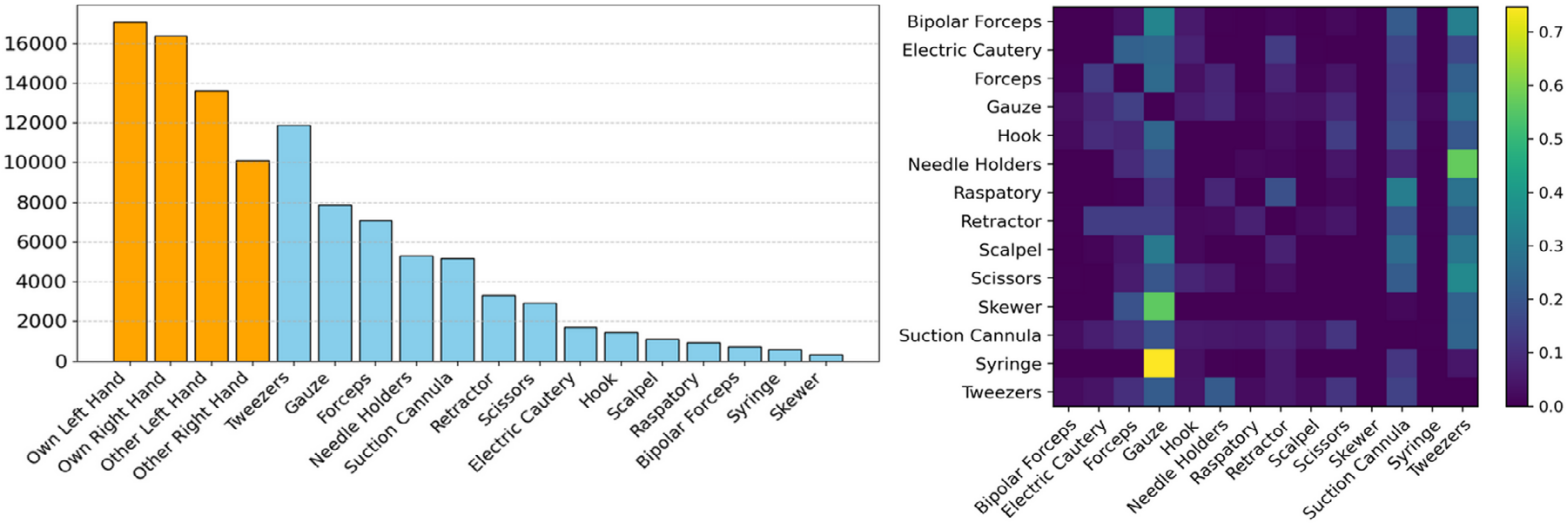
Dataset's tools and hands distribution and tools co‐occurrence matrix. (Left) Distribution of different hands and surgical tools instances. (Right) Frame level co‐occurrence matrix tools in the dataset.

**FIGURE 3:**
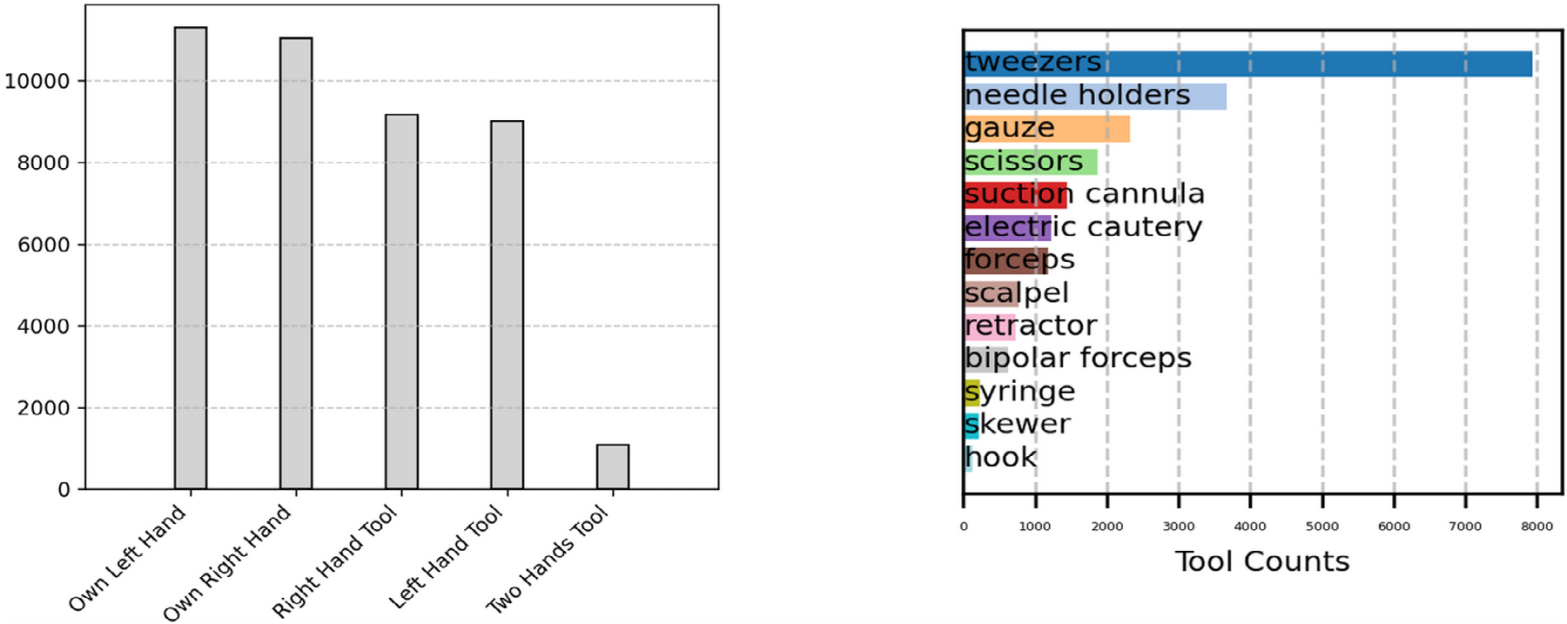
Hand‐tool interaction statistics. (Left) Distribution of hands and its associated tools. (Right) Tool usage counts based on manual handling.

The EgoSurgery‐HTS dataset is composed of 15 videos from the original 20 EgoSurgery videos, specifically the same 15 that were annotated with bounding boxes in EgoSurgery‐Tool. In this subset, 2 videos of serial resection of skin lesion, 5 videos of skin tumour resection, 1 video of posterior pharyngeal flap, 1 video of subcutaneous tumour resection, 4 videos of alveolar bone grafting, 1 video of scar revision and 1 video of open reduction and internal fixation are represented for a total of 7 different types of surgeries. The dataset is composed with 18,450 high‐quality annotated images, with 48,106 tools, 55,655 hands, and 39,578 hand–tool segmentations. Figure 2 (left) illustrates a pronounced class imbalance among the different surgical tools. To further analyse tool co‐occurrence patterns, we introduce a co‐occurrence matrix in Figure 2 (right), which shows the probability of finding one tool given the presence of another. Notably, certain tools frequently appear together, such as syringes and gauze, or needle holders and tweezers. This highlights strong tool pairings like Syringe/Gauze and Needle Holder/Tweezers. Figure 3 focuses on hand–tool segmentation, showcasing the distribution of hands and their associated tools. Additionally, it provides insights into the frequency of tool usage within the dataset. Tweezers and needle holders are used significantly more often than other surgical tools, indicating an uneven distribution of tool usage across surgical operations.

## 3 Experiments and Benchmarking Methods

### 3.1 Experimental Setups

We evaluate four popular object detectors: Mask R‐CNN [31], QueryInst [32], Mask2Former [33] and SOLOv2[34] on our dataset. The implementations are based on the MMDetection [35], and we finetune models pre‐trained on the MS‐COCO [36] dataset. To ensure a fair comparison, we select model backbones with a similar number of parameters. Following the original EgoSurgery‐Tool methodology, we adopt the exact same videos and video‐split for model training, considering the domain variations across videos, using 10 training, 2 validation and 3 testing videos. Unlike random frame‐level splits, this method ensures that entire videos are reserved for either training or testing, which is critical in reducing data leakage and preventing models from memorizing context‐specific features unique to a particular surgery. This setting promotes domain generalization in open surgical contexts. Moreover, we also further distinguish between the segmentation task of all tool instances and all hand instances, as each task presents distinct characteristics and requirements. Treating them as separate tasks is therefore more appropriate than addressing them jointly.

### 3.2 Evaluation Metrics

The experimental results are reported using the standard COCO metrics, where the average precision (AP) is computed as the mean intersection over union (IoU) across 10 thresholds ranging from 0.5 to 0.95 (at intervals of 0.05) for both bounding boxes (box AP) and segmentation masks (mask AP).

### 3.3 Quantitative Results

#### 3.3.1 Surgical Tool Segmentation

**FIGURE 4:**
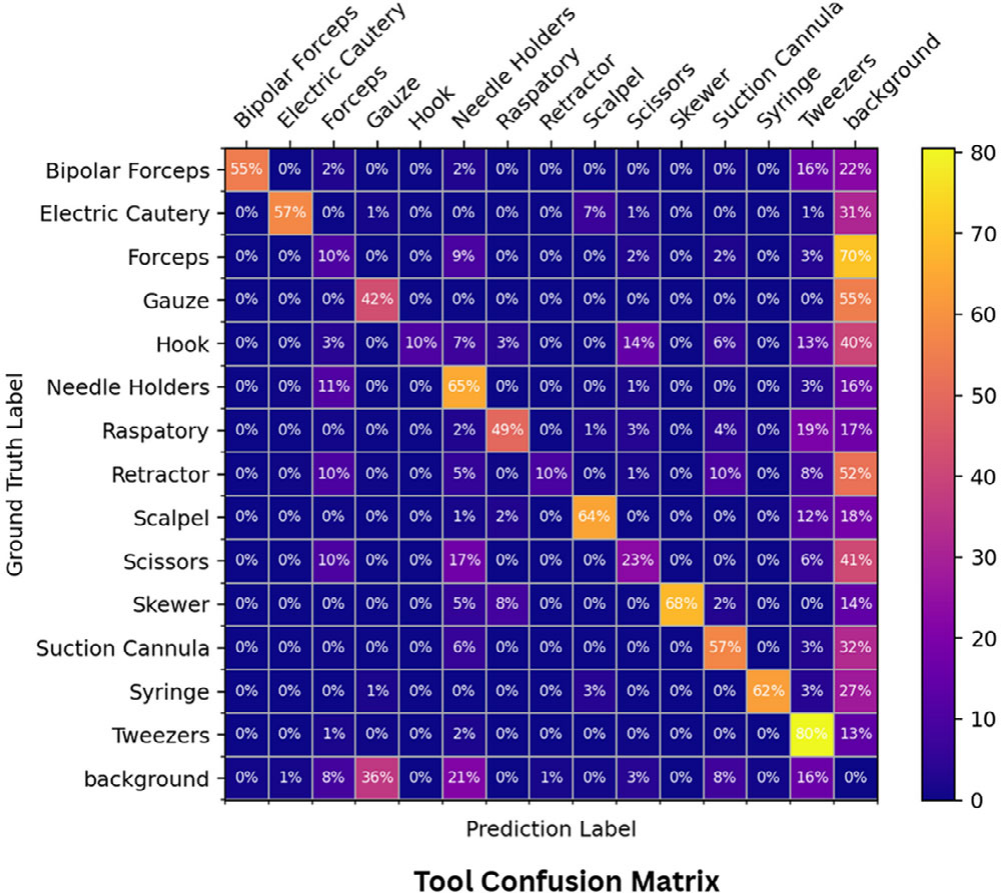
Mask2Former confusion matrix for the tool segmentation task, illustrating correct predictions on the diagonal and main misclassification patterns across tool classes.

For surgical tool detection, the box mAP are 36.7%, 47.3% and 39.2% and the segmentation mAP are 29.1%, 36.7%, 40.9% and 37.0% for the respective Mask R‐CNN, QueryInst, Mask2Former and SOLOv2 models. The experimental results are presented in Table 2. QueryInst achieves the highest performance in terms of the mAP metric for surgical tool detection tasks with bounding box but Mask2Former outperforms the rest of the models in terms of mAP for per‐pixel surgical tool segmentation. While Mask2Former demonstrates superior performance in tool segmentation tasks, the specific reasons behind this improvement in the context of open surgery remain unclear. Further experiments would be required to determine whether factors such as its global attention mechanisms or other design choices contribute most significantly to its effectiveness in open surgery context. In addition, we observe some confusion in the models for tools with similar shapes in Figure 4. Moreover, each unique tool's average precision strongly depends on the tool's appearance frequency. The unbalance in tool's appearance offers a great disparity in mAP prediction for each tool. It leads to underperforming models induced by a lack of data on certain tools. The overall results need to be improved, but similar to EgoSurgery‐Tool results [27, 28], the results are encouraging for the future of practical tool segmentation in the context of open surgery.

**TABLE 2:** Experimental results (%) of four state‐of‐the‐art models using a ResNet‐50 backbone on three tasks: tool instance segmentation, hand instance segmentation, and hand–tool segmentation.

	Tool		Hand		Hand–Tool	
Methods	${\text{mAP}}^{\text{box}}$	${\text{mAP}}^{\text{mask}}$	${\text{mAP}}^{\text{box}}$	${\text{mAP}}^{\text{mask}}$	${\text{mAP}}^{\text{box}}$	${\text{mAP}}^{\text{mask}}$
Mask R‐CNN [31]	36.7	29.1	**63.8**	**61.9**	45.3	44.5
QueryInst [32]	**47.3**	36.7	54.0	50.7	**55.2**	49.4
Mask2Former [33]	39.2	**40.9**	50.2	52.5	54.7	**56.6**
SOLOv2 [34]	—	37.0	—	53.8	—	50.7

#### 3.3.2 Hand/Hand–Tool Segmentation

**FIGURE 5:**
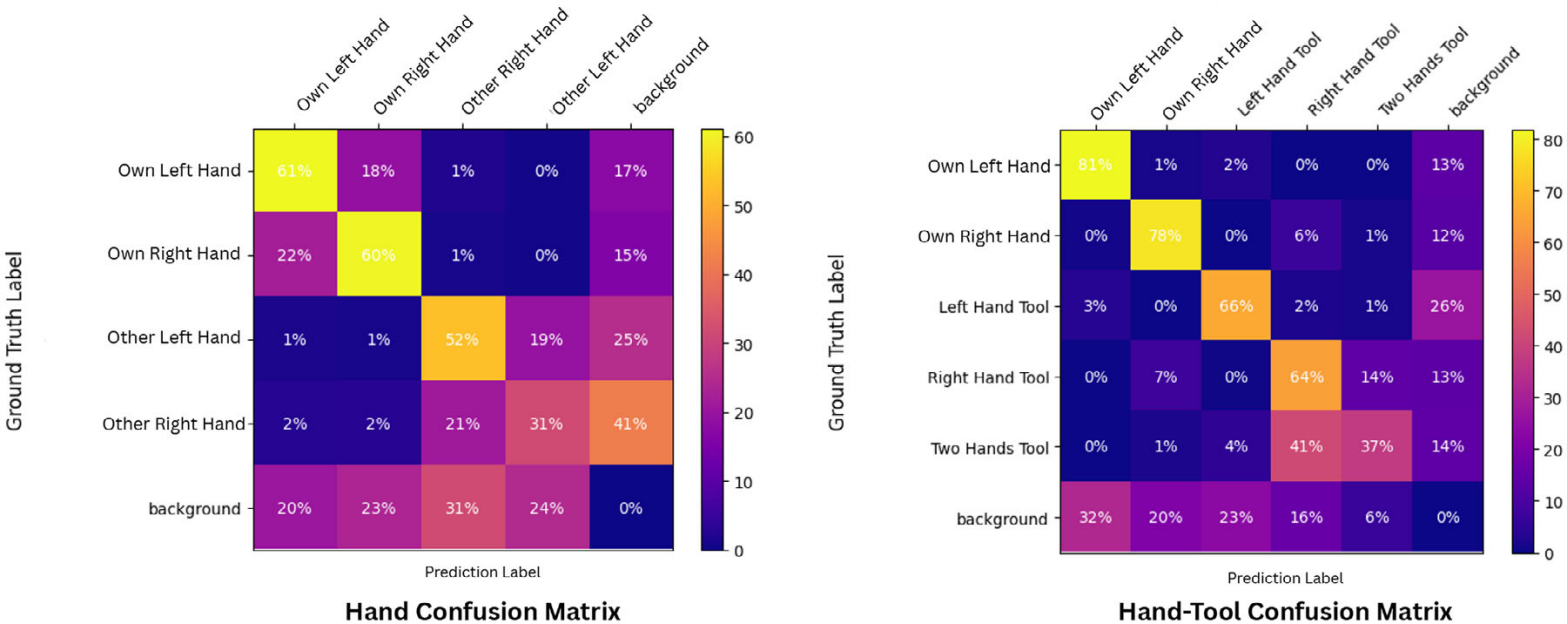
Mask2Former confusion matrix over hand and hand‐tool segmentation task. (Left) hand confusion matrix. (Right) hand‐tool confusion matrix.

The benchmark results presented in Table 2 for hand segmentation highlight great performances of Mask R‐CNN with a box mAP of 63.8% and a mask mAP of 61.9%. However, Mask R‐CNN seems underperformant in surgical tool segmentation tasks compared to the three others. Mask2Former consistently outperforms Mask R‐CNN, QueryInst and SOLOv2 in segmentation mAP. For bounding box mAP in hand–tool detection, QueryInst achieves the best performance. Out of the four models overall, Mask2Former is superior if tool segmentation is part of the task. In the case of only hand segmentation, Mask R‐CNN outperforms the other three models. Figure 5 reveals a higher degree of confusion among other hands, which are less distinct and less precisely defined compared to the owner's hands. Nevertheless, these tasks performances clearly show promising results for real applications in open surgical operations.

#### 3.3.3 Comparison with Other Dataset

We compare QueryInst Hand and Hand–Tool segmentation performance on our testing set for different training data. Comparison results are presented in Table 3 for Hand and Hand–Tool segmentation task. Regarding Hand Segmentation, training on our dataset significantly outperforms training on EgoHands. Similarly, we observe superior results when training on our dataset as opposed to the Kitchen VISOR dataset. This strongly indicates a domain transfer issue from the representation of hands and tools in daily activities to open surgical ones. Our dataset serves as a valuable asset, enabling the learning of novel representations of surgical objects in the challenging environment of open surgery.

**TABLE 3:** Performance comparison of QueryInst trained on EgoSurgery‐HTS and model trained on existing hand segmentation and hand–tool segmentation datasets.

(a) Hand segmentation			(b) Hand–tool segmentation		
Dataset	${\text{mAP}}^{\text{box}}$	${\text{mAP}}^{\text{mask}}$	Dataset	${\text{mAP}}^{\text{box}}$	${\text{mAP}}^{\text{mask}}$
EgoHands [23]	8.3	6.3	VISOR‐HOS [21]	13.0	11.4
Grey EgoSurgery‐HTS	**54.0**	**50.7**	Grey EgoSurgery‐HTS	**55.2**	**49.4**

### 3.4 Qualitative Results

**FIGURE 6:**
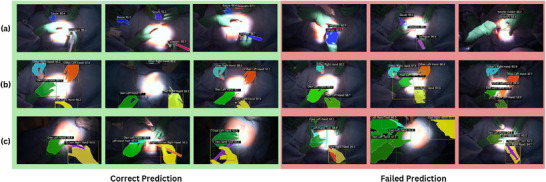
Overview of the Mask2Former model predictions on the three tasks. (a) Tool segmentation task. (b) Hand segmentation task. (c) Hand‐tool segmentation task.

The qualitative performance of the Mask2Former model from the baseline is illustrated in Figure 6. The model demonstrates its capability to accurately segment surgical tools, hands, and hand–tool interactions across a wide variety of surgical procedures.

This suggests that it generalizes well despite the inherent variability in open surgery environments. However, we still observe notable failure cases, including missed detections, segmentation errors, and hallucinated instruments in particularly challenging frames. These issues often arise in scenes with extreme lighting conditions, strong shadows, low contrast, complex occlusions, and ambiguous tool shapes or textures which are factors that are common in real‐world open surgery footage.

**FIGURE 7:**
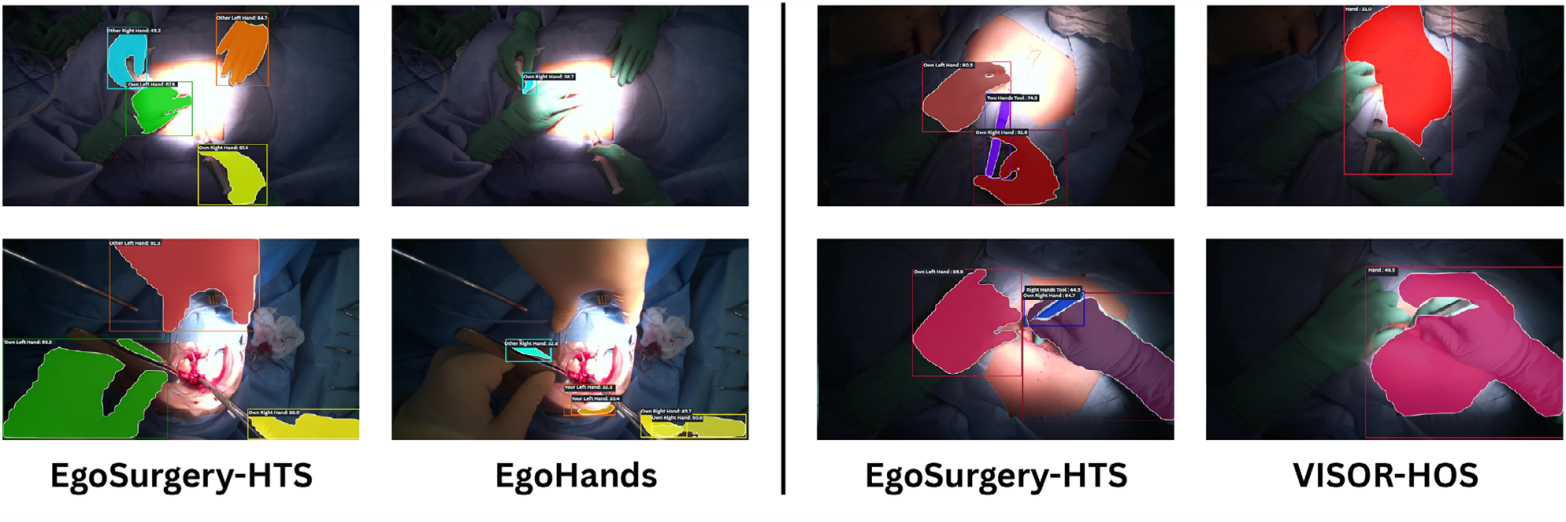
Qualitative predictions of QueryInst trained on EgoSurgery‐HTS dataset versus trained on Egohands (left) for hand segmentation and trained on VISOR‐HOS (right) for hand–tool segmentation task.

Despite these challenges, the model exhibits strong robustness, maintaining consistent performance even in suboptimal or visually complex scenarios. This suggests that the model has effectively learned to capture the most salient features required for distinguishing surgical instruments and hands in dynamic and unstructured environments. Moreover, the results highlight a substantial performance boost when the model is trained on our proposed dataset compared to previous publicly available ones. This is especially evident in the context of open surgery, where traditional datasets fall short in representing the visual and contextual complexity of real‐life operating rooms. These improvements are clearly visible in the comparative results shown in Figure 7, underscoring the relevance and value of our dataset for advancing segmentation models in surgical settings.

## 4 Conclusion and Future Work

In this paper, we introduce EgoSurgery‐HTS, the first egocentric open‐surgery segmentation dataset, including all hands and surgical tool instances plus the association of hand and tool. The dataset is composed of raw surgical videos of surgery with extensive segmentation annotations of every instance present. We define three tasks to improve the understanding of the egocentric open surgery scene through our dataset: hand segmentation, tool segmentation, and hand–tool segmentation. We demonstrated the benefit of using EgoSurgery‐HTS compared to other egocentric hand–tool datasets through pretrained model evaluations. A benchmark for each task is proposed as a reference for future evolution of the models evaluated on this dataset. We validate the utility of EgoSurgery‐HTS by benchmarking several pretrained models and comparing their performance against those trained on other egocentric hand–tool datasets, demonstrating its superior capacity to enhance generalization in open‐surgery contexts. Finally, we establish a comprehensive benchmark for each task, providing a strong baseline and a foundation for future research aimed at advancing perception models in surgical segmentation.

Despite promising results, mAP results are still not acceptable for critical surgery applications. The continuing hurdles are to improve model for better segmentation to become usable during real case application. An auspicious next step is to focus on minimizing the challenges of segmenting heavily imbalanced data. Upcoming research efforts will concentrate on collecting more tool instances to equilibrate the tool distribution and increase the robustness of the model through additions of different egocentric open surgery environment.

## Author Contributions

**Nathan Darjana**: conceptualization, data curation, investigation, methodology, software, writing – original draft, writing – review and editing. **Ryo Fujii**: conceptualization, data curation, investigation, methodology, software, supervision, writing – review and editing. **Hideo Saito**: conceptualization, funding acquisition, investigation, project administration, supervision, writing – review and editing. **Hiroki Kajita**: conceptualization, funding acquisition, investigation, methodology, project administration, supervision.

## Funding

This research is supported by Japan Society for the Promotion of Science Grants‐in‐Aid for Scientific Research B 25K03143.

## Conflicts of Interest

The authors declare no conflicts of interest.

## Data Availability

The data that support the findings of this study are available from the corresponding author upon reasonable request.
